# Genetic and Environmental Contributions to Weight, Height, and BMI from Birth to 19 Years of Age: An International Study of Over 12,000 Twin Pairs

**DOI:** 10.1371/journal.pone.0030153

**Published:** 2012-02-08

**Authors:** Lise Dubois, Kirsten Ohm Kyvik, Manon Girard, Fabiola Tatone-Tokuda, Daniel Pérusse, Jacob Hjelmborg, Axel Skytthe, Finn Rasmussen, Margaret J. Wright, Paul Lichtenstein, Nicholas G. Martin

**Affiliations:** 1 Institute of Population Health, University of Ottawa, Ottawa, Ontario, Canada; 2 Department of Epidemiology & Community Medicine, University of Ottawa, Ottawa, Ontario, Canada; 3 Institute of Regional Health Services Research, University of Southern Denmark, Odense, Denmark; 4 Odense Patient Data Explorative Network, Odense University Hospital, Odense, Denmark; 5 Faculté des Arts et des Sciences, Université de Montréal, Montreal, Quebec, Canada; 6 Department of Biostatistics, University of Southern Denmark, Odense, Denmark; 7 Department of Public Health Sciences, Karolinska Institute, Stockholm, Sweden; 8 Queensland Institute of Medical Research, Brisbane, Australia; 9 Department of Medical Epidemiology and Biostatistics, Karolinska Institute, Stockholm, Sweden; Johns Hopkins Bloomberg School of Public Health, United States of America

## Abstract

**Objective:**

To examine the genetic and environmental influences on variances in weight, height, and BMI, from birth through 19 years of age, in boys and girls from three continents.

**Design and Settings:**

Cross-sectional twin study. Data obtained from a total of 23 twin birth-cohorts from four countries: Canada, Sweden, Denmark, and Australia. Participants were Monozygotic (MZ) and dizygotic (DZ) (same- and opposite-sex) twin pairs with data available for both height and weight at a given age, from birth through 19 years of age. Approximately 24,036 children were included in the analyses.

**Results:**

Heritability for body weight, height, and BMI was low at birth (between 6.4 and 8.7% for boys, and between 4.8 and 7.9% for girls) but increased over time, accounting for close to half or more of the variance in body weight and BMI after 5 months of age in both sexes. Common environmental influences on all body measures were high at birth (between 74.1–85.9% in all measures for boys, and between 74.2 and 87.3% in all measures for girls) and markedly reduced over time. For body height, the effect of the common environment remained significant for a longer period during early childhood (up through 12 years of age). Sex-limitation of genetic and shared environmental effects was observed.

**Conclusion:**

Genetics appear to play an increasingly important role in explaining the variation in weight, height, and BMI from early childhood to late adolescence, particularly in boys. Common environmental factors exert their strongest and most independent influence specifically in pre-adolescent years and more significantly in girls. These findings emphasize the need to target family and social environmental interventions in early childhood years, especially for females. As gene-environment correlation and interaction is likely, it is also necessary to identify the genetic variants that may predispose individuals to obesity.

## Introduction

The global obesity epidemic is accelerating [1] and has affected virtually all ages, races, and sexes in developed and developing countries [2], [3]. The obesity increase in childhood is especially troubling as overweight/obesity is shown to track into later adolescent and adult years [4] and is associated with numerous immediate and long-term health risks that lead to morbidity and premature mortality (e.g. asthma, type 2 diabetes, cardiovascular diseases, and cancer) [1].

Overweight/obesity has a multifactorial aetiology; moreover, there has not been a substantial change in mankind's genetic makeup to explain the obesity epidemic that has ravaged the world over the last three decades [5].This epidemic is mainly attributed to a global shift in the consumption of calorie-dense diets and reduced physical activity, a trend that has accompanied globalization and is further exacerbated by various individual, societal, and socioeconomic factors [3], [6], [7]. Nonetheless, not all individuals exposed to obesogenic environments become obese. A genetic propensity for weight gain and obesity must be present for the environment to precipitate an overweight/obese phenotype. Twin, family, and adoption studies provide strong evidence for large genetic influences on variations in body mass index (BMI), with heritability estimates ranging from 50% to over 90%, leaving the remaining variance attributed to environmental influences, whether common to family members/siblings or unique to the individual [8]–[10]. However, these estimates have varied widely across studies due to differences in study types, populations, and ages targeted.

Twin studies generally provide higher heritability estimates in comparison to adoption and family studies, and they are considered to provide the most precise estimates of the genetic and environmental influences on behavioural and physical phenotypes [8]. Most large-scale twin studies involve adult populations, and these show a very small to no effect of the common environment on variations in BMI [9], [11]; rather, it is the unique environment that generally influences the remaining variance in BMI in adulthood. The role of the common environment may be more significant in childhood, however, as there are more frequent opportunities for twins to be exposed to the same environmental influences while living together with parents and other siblings. In fact, a recent systematic review and meta-analysis of twin and adoption studies found that common environmental factors showed a substantial influence on variations in BMI in mid-childhood, although this influence vanished in adolescence between the ages of 14 and 17 years [10]. A review of twin and family studies also observed that, while there are strong genetic influences on the tracking of BMI from early childhood to the beginning of adulthood, there is also evidence that common environmental influences are important throughout childhood [12]. However, much of research on child and adolescent twins is limited to specific local populations, fairly small samples, or only examines a portion of childhood and adolescence. The critical years where interventions can be made to target common and unique environmental influences on these body measures have not been identified as, to date, no large-scale twin study has yet examined the genetic and environmental influences on variances in body weight, height, and BMI over each year of age, from birth to adulthood.

Little is also known about sex-differences in the heritability of body weight, height, and BMI from birth to late adolescence. It is well known that girls in general mature more rapidly than boys, but only a few studies have examined sex-limitation in genetic and environmental influences on variations in these body measures over childhood and adolescence; these show inconsistent results. One study in twins aged 8–11 years found no evidence of sex-limitation in the heritability of BMI or waist circumference [13]; whereas, other studies report age-specific sex-differences in twins at 5 months [14], between 16 and 17 years, [15] and between 18 and 25 years [16]. Furthermore, where sex-differences are observed, it is unclear which sex is more strongly influenced by genetic factors for the variability of various anthropometric measures. Some studies report higher heritability estimates in body weight, height, or BMI for females [14], [16]–[18], whereas others report stronger genetic influences in males [19]. Further large-scale studies on child and adolescent monozygotic (MZ) and dizygotic (DZ) twin pairs are needed to clarify these inconsistencies.

Using international, population-based data obtained from large twin birth-cohorts in three different continents, the present study aims to describe the distribution of weight, height, and BMI in MZ and DZ same- and opposite-sex twin pairs, from birth through 19 years of age, and examine the genetic and environmental influences on variances in these body measures over each year of age during the first 19 years of life; sex-limitation of genetic and environmental effects will also be explored at each age examined.

## Methods

### Ethics Statement

Ethics approval was obtained for each of the respective cohorts and participants gave informed consent.

### Data Sources

The present study analyzed data obtained from a total of 23 twin birth-cohorts from four different countries: Canada, Sweden, Denmark, and Australia. Analyses included data from MZ and DZ (same- and opposite-sex) twin pairs with available measures for both height and weight at a given age, from birth through 19 years of age. From an initial sample of approximately 30,500 children, 24,036 children provided data for the analyses. A brief overview of the cohorts from which the study samples were drawn is provided in the following subsections. Sample sizes and characteristics of children included in the present study are provided in Table S1.

#### From Canada: ‘Québec Newborn Twin Study’ (QNTS)

The Québec Newborn Twin Study (QNTS) [20] is a population-based birth cohort of twin births occurring between April 1^st^, 1995 and December 31^st^, 1998 in the seven health districts of the greater Montreal area in the province of Québec, Canada. Out of a total of 989 families contacted, 672 agreed to participate (68%). Twins with chronic diseases and those who died prior to the age of 5 months were excluded from the cohort. The twins were first seen between the ages of 59 and 61 weeks (or 5 months, corrected for gestational age); these were followed annually thereafter. Each year, parents provided their informed consent.

Zygosity was determined when twins were 5 and 18 months old through the aggregation of independent tester ratings based on live assessments of physical similarity between twins; this was accomplished using a shortened version of the Zygosity Questionnaire for Young Twins [21]. At ages 5 and 18 months, mouth swabs were also collected from a random subsample of same-sex twins; DNA was extracted from the cells, amplified by polymerase chain reaction, and typed using 8 to 10 highly polymorphic micro-satellite markers. A comparison between physical assessments and genotyping yielded a 91.9% concordance among a random subsample of 123 pairs assessed at 5 months of age, and a 93.8% concordance among a subsample of 113 pairs assessed at 18 months [22]. With consideration for chorionicity data obtained from the twins' medical files, 96% twin pairs were thus classified correctly.

Anthropometric measures of children's weights (in grams; g) and heights (in centimeters; cm) were taken at birth (drawn from medical records), at 5 months, and at 5 and 8 years through laboratory measures. In order to eliminate potential biases related to perceived zygosity, different research assistants took the measures for each child within a twin pair.

#### From Sweden: ‘Child and Adolescent Twin Study in Sweden’ (CATSS) and ‘Twin Study of Child and Adolescent Development’ (TCHAD)

From Sweden, three different cohorts of children and adolescents were used for the analyses: two from the *Child and Adolescent Twin Study in Sweden (CATSS)* and one from the *Twin Study of Child and Adolescent Development (TCHAD)* [23]. Both studies are based on twins included in the Swedish Twin Registry (STR) [24].

The Child and Adolescent Twin Study in Sweden (CATSS) [24] started in September 2004 and it is an ongoing study that includes twins born between 1992 and 2001. Zygosity determination for 571 pairs of twins in whom DNA from both twins was available was based on a panel of 48 single-nucleotide polymorphisms derived for zygosity analyses [23]. For the remaining twins, an algorithm based on 5 items concerning twin similarity and confusion [24] derived from the twins with known zygosity was used. Only twins with more than 95% probability of being correctly classified were assigned a zygosity. In this study, parents of all Swedish twins turning 9 or 12 years were approached to complete a telephone interview regarding various health and behavioural issues about their twin children. Certain families were followed to complete additional questionnaires, genotyping, and further clinical interviews. To date, the survey holds an 80% response frequency, with 7,408 interviews completed by November 2008. Twins' birth-weights, lengths, and heights and weights at age 9 and 12 years are parent-reported and obtained through telephone interviews.

The Twin Study of Child and Adolescent Development (TCHAD) [23] follows 1,480 twin pairs from ages 8 to 20 years. Twins included in the study were those born in Sweden between May 1985 and December 1986. Zygosity was determined by using discriminant analyses on 385 twin pairs with known zygosity which were confirmed by 47 polymorphic DNA-markers [23]. This algorithm is restricted to classify monozygotic twins (MZ) and dizygotic twins (DZ) with 95% accuracy [25]. A questionnaire of four items covering the twins' physical similarities were answered at age 8–9 (via parent-reports) and at age 13–14 and 16–17 years (via both parent- and self-reports). Zygosity classification was made for each response separately through discriminant analysis. A final zygosity assignment was set if there were no disagreements between the five separate assignments. In cases of any contradictions between the assignments, the zygosity score was set to ‘unknown’.

The study was conducted in four waves, starting in 1994 (when the twins were 8–9 years old), then again in 1999 (at age 13–14 years), in 2002 (at age 16–17 years), and in 2006 (at age 19–20 years). Questionnaires were administered to the parents and twins over the telephone. Twins' birthweights were obtained from the Swedish Medical Birth Register. Measures for twins' heights and weights at later ages were parent-reported in the first questionnaires at age 8–9 years, and were both parent- and self-reported by each twin in the following study waves. The response rates for the four study waves were: 91% (*n* = 1339 parents) in Wave 1 for the parent-questionnaires; 73% (*n* = 1063 parents) and 78% (*n* = 2263 twins) in Wave 2 for the parent- and twin- questionnaires, respectively; and 74% (*n* = 1067 parents) and 87% (*n* = 2369 twins) in Wave 3.

#### From Denmark: ‘Danish Twin Registry’ (DTR)

For the present study, data from a total of 18 birth cohorts of twins born in 1983–2000 were obtained from the Danish Twin Registry [26]. The DTR is an ongoing population-based twin registry that initiated in 1954 and, by the end of 2005, included over 75,000 twin pairs born between 1870 and 2004 [27]. Zygosity in the DTR is determined through questions examining the degree of similarity between same-sex co-twins, which has been validated by DNA finger printing and found to be correct in more than 97% of cases [28]. The present cohort was approached with the Danish Twin Child Survey in 2003: a short questionnaire was administered to the parents if the twins were born in 1988–2000 and to the twins themselves if they were born in 1983–87. The questionnaire included questions on weight and height at birth and at the age of the twins when answering in 2003 (i.e. from ages 3 to 19 inclusively). A total of 29,711 twin individuals were approached and 19,782 (66.6%) provided answers.

#### From Australia: Brisbane Longitudinal Twin Study (BTLS)

The data from this sample were collected through the ongoing Brisbane Longitudinal Twin Study (1992–2010) where twins are evaluated for melanoma risk factors at ages twelve and fourteen [29], [30], and for cognitive variables at age sixteen [31]. Participants are ascertained from schools in south-east Queensland and are of mainly European extraction, most with Anglo-Celtic ancestry. Blood samples are obtained for zygosity confirmation and DNA extraction. At each visit, height is measured with a stadiometer and weight is measured using frequently recalibrated scales.

### Measures

Data on weight (in kilograms, Kg), height (in meters, m), and BMI (kg/m^2^) from birth through 19 years of age were standardized to z-scores with a mean of zero and a standard deviation of one. The test for normality was employed to ensure that data were normally distributed (i.e., no transformation was needed). Furthermore, data were adjusted for repeated measurement (in SAS, the REPEATED statement controls the covariance structure imposed on the residuals or errors). Where data were available from more than one cohort or more than one country for a given age, they were pooled (data from 6 datasets were used in multivariate analyses). The proportions that twin pairs represent are given by dataset, age, zygosity, and sex (Table S1) after confirming that cohort distributions were similar (data not shown). No family outliers (i.e., bivariate outliers) exceeding three standard deviation from the mean were identified using the mahalanobis distance for each family represented as a Z-score.

### Statistical Analyses

Intra-class correlations were computed for five zygosity-by-sex groups (MZ-boys, MZ-girls, DZ-boys, DZ-girls, DZ-opposite-sex) and for the total number of MZ and DZ twin pairs included in the study. MZ twins, being genetically identical, share 100% of their segregating genes, whereas non-identical DZ twins share on average 50%. For this reason, if phenotypic variation in a specific trait is due to genetic effects, more resemblance in that trait will be found within MZ twins in comparison to DZ twins. However, two important assumptions must be made: Firstly, the environment to which each twin in a MZ and DZ pair is exposed is assumed to be similar; and secondly, results for the genetic and environmental influences on phenotypic variation in twins is assumed to be generalizable to singletons in the rest of the population. Several publications have discussed these two assumptions [32]–[34].

Classical model-fitting techniques [17] were used to test for different models and to quantify the magnitude of the genetic and environmental influences on variations in the body measures. As twins form a natural two-level hierarchy, a hierarchical random-effect multilevel model of twin data [35] that allows for a full likelihood estimation of all parameters [36] was used. A model was first built by specifying means, between-pair, and within-pair variances separately for MZ and DZ twins [14]; doing so equates the predicted means, variances, and covariances of the model to their observed values in both twin groups. The conditions of equal means and variances of MZ and DZ twins were imposed (as well as for twin A and twin B). In a twin study, the random part of this model can be specified to reflect four components of phenotypic variation in a specific trait: 1) *additive genetic* (A) variation, the sum of the effect of all alleles on a specific trait over all loci; 2) *non-additive genetic* (D) variation, the non-additive effect of alleles in the same locus with the inclusion of dominance genetic effects, caused by interactions between alleles in the same locus, and epistasis (interactions between alleles at different loci); 3) *common environmental* (C) variation, which consists of environmental factors shared by twins; and, 4) *unique environmental* (E) variation, which consists of environmental factors that are unique to each individual and includes measurement error. When analyzing information on twins reared together, the C and D components cannot be estimated simultaneously [37], [38]. Thus, one can estimate four parameters in a resulting ACE (or ADE) model with two degrees of freedom: A phenotypic mean, additive genetic variance, common environmental variance (or non-additive genetic variance), and unique environmental variance. Specifications can also be formulated to examine submodels, including: 1) a CE model that removes all genetic components (suggesting no genetic effect); and, 2) an AE model that suggests no effect of the common/family environment. These are all considered to be univariate models. The square of path coefficients (i.e. a^2^, c^2^ or d^2^, and e^2^) or variance components are typically used to express the expected variances and covariances between individuals in twin pairs. These values are calculated using matrix algebra to identify the A, C (or D), and E components, respectively.

Generally, factors that constitute C, ‘common environmental influences’, in childhood and early adolescence that relate to body weight and BMI include: family's socioeconomic status [39] parenting style and parental modeling of healthy eating and activity behaviours [40], the home, school, and community food environment [41], [42], and neighbourhood characteristics [43]. Examples of E, ‘unique environmental influences’, include exposure to a virus or an injury/accident, among others. Given that data at several ages were pooled from two or more of the countries included in the present study sample, we have chosen to focus primarily on ACE (instead of ADE) models (i.e., to examine C rather than D) because the magnitude of the MZ and DZ same-sex intraclass correlation ratios tend to satisfy inequality [2r_DZ_>r_MZ_>r_DZ_] at different ages, thus evidencing common (shared) genetic influences (Table S5). Therefore, examining components A, C, and E yields a broader portrait of the genetic and environmental influences on weight, height, and BMI in this international population.

**Figure 1:**
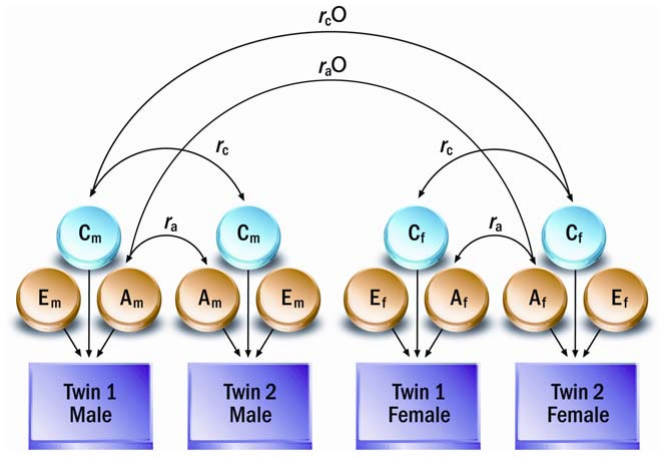
General sex-limited model. The m and f subscripts refer to males and females, respectively. r_a_ and r_d_ are additive genetic and common environmental correlations between same sex twins; r_a_O and r_c_O are additive genetic and common environmental correlations between opposite sex twins.

Using these classical methods, the twin design can also be extended to examine sex-limitation in the genetic and/or common environmental influences on the variability in a specific trait. This is accomplished by testing two models (one model per sex) simultaneously, while controlling for the covariance between opposite-sex DZ twins in A and C components. Including opposite-sex DZ twins in these analyses increases power and permits one to examine an additional male or female additive genetic (A′_F_ or A′_M_) or common environmental (C′_F_ or C′_M_) component that does not correlate with the genetic or environmental influences observed in the phenotype displayed on the female or male counterpart (Figure 1). Thus, ACE, CE, and AE models can be examined all with common effects, correlated effects, and uncorrelated effects. Observing significant estimates in a sex-limitation model provides evidence that the genetic or environmental factors that influence variability in a trait are not identical across sexes. Further detail pertaining to sex-limited modeling techniques is available elsewhere [17].

For each dependent variable and for each age examined in the present study, a sex-limited saturated model and a univariate saturated model were first fitted to examine a sex-effect in the genetic component. Using the likelihood ratio test (−2Log), the resulting two saturated models were then compared; as this test was shown to be significant at the 0.05 level for almost all models at every age (except for weight and height at age 4 and 7 years, and BMI at 9 years), the sex-limited models were used in the analyses at all ages. Subsequent nested models were examined, beginning from saturated to reduced models (ACE, AE, CE, and E). Nested models were compared to the full saturated models using a likelihood ratio test (−2Log) and Akaike's Information Criterion (AIC: chi-square-2*df*) which also considers both goodness-of-fit and parsimony in a model's explanatory value. Selecting a model based on the AIC tends to produce more power. All statistical analyses and model-fitting were conducted using SAS/NLMIXED 8.2 and statistical significance was set at 0.05.

## Results

Data on weight, height, and BMI were available from all 23 cohorts at birth, from two cohorts (from two different countries) at ages 5, 8, 9, 13, and 14 years, and from three cohorts (from three different countries) at ages 12 and 16 years; data were obtained from single cohorts for all remaining ages (at 5 months, and at ages 3, 4, 6, 7, 10, 11, 15, and 17 to 19 years, inclusively). Data at ages 1 and 2 years were not available from any of the cohorts included in the present study.

**Figure 2:**
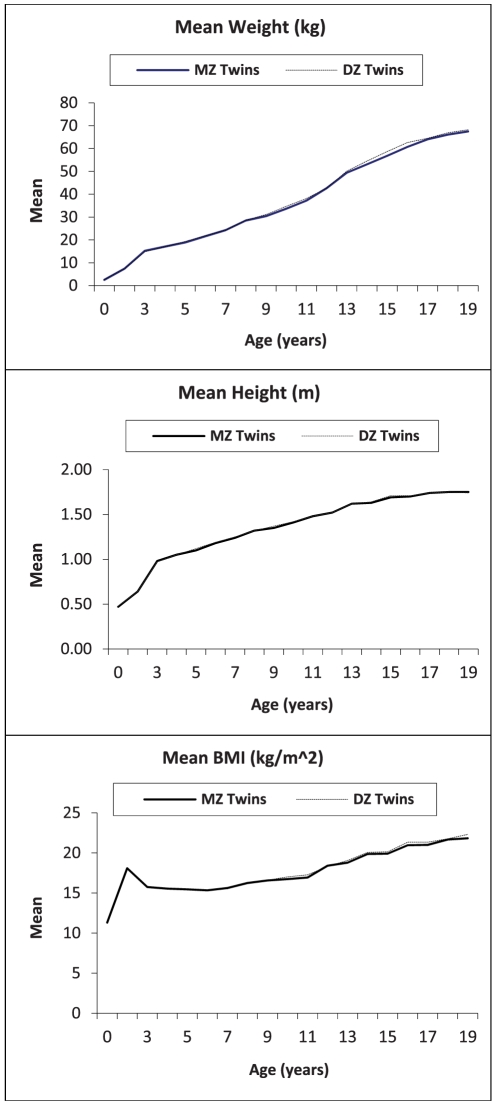
Mean of weight (kg), height (m), and BMI (kg/m^2^) in MZ and DZ twins of four countries, from birth through 19 years of age.

**Figure 3:**
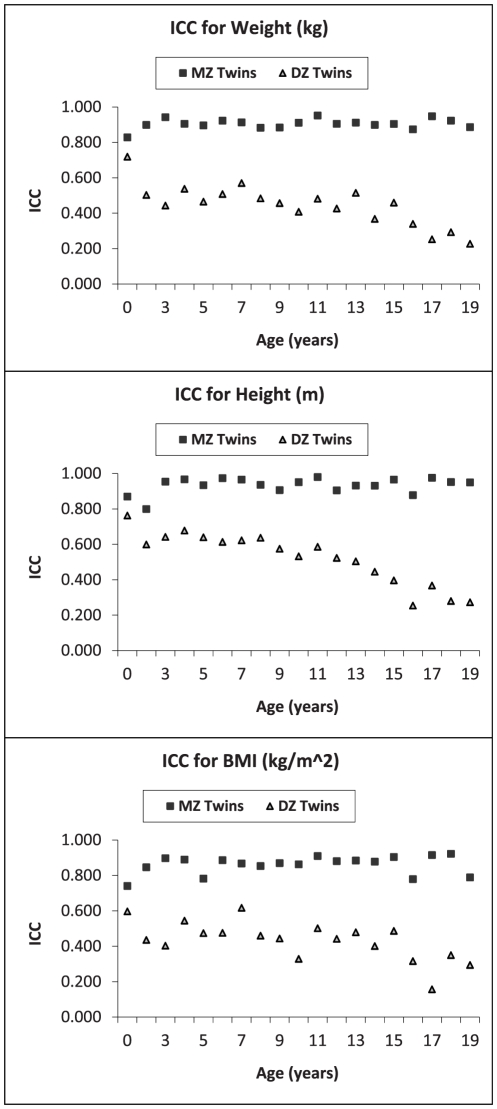
Intra-class correlations (ICC) between MZ and DZ twin pairs for weight (kg), height (m), and BMI (kg/m^2^), from birth through 19 years of age.

The mean birthweight for all children included in analyses, irrespective of zygosity and sex, was 2.6 kg (SEM = 0.00). Mean weight, height, and BMI values from birth through age 19 years for MZ and DZ twins from all cohorts combined are presented in Figure 2 (the *number* of MZ and DZ twins included at each age, along with means and standard errors for weight, height, and BMI are available in Tables S2, S3, and S4, respectively). Over all ages, MZ and DZ twins maintained similar patterns of growth in mean weight, height and BMI, with a sharp increase in growth from birth to age 3 years, and then a steady increase in both weight and height through 19 years of age. When intra-class correlations between MZ and DZ twins were examined (Figure 3), irrespective of sex, MZ twin correlations were consistently greater than those of DZ twins for weight, height and BMI, and the gap between MZ and DZ correlations increased over time. While MZ twins maintained a correlation of approximately 0.8 or greater in weight, height, and BMI from birth through age 19 years, DZ twin correlations in weight reduced from around 0.7 to close to 0.2 by 19 years of age, and from around 0.8 to approximately 0.3 for height over those same years, indicating the presence of strong genetic effects. Sex-specific correlations between MZ and DZ twins were also examined for weight, height and BMI. At all ages examined, and for all measures (except for height at age 5 months), intraclass correlations for MZ twins by sex differed significantly from DZ twins of the same sex (data available in Table S5), suggesting possible sex-limitation in the heritability of these body measures.

**Figure 4:**
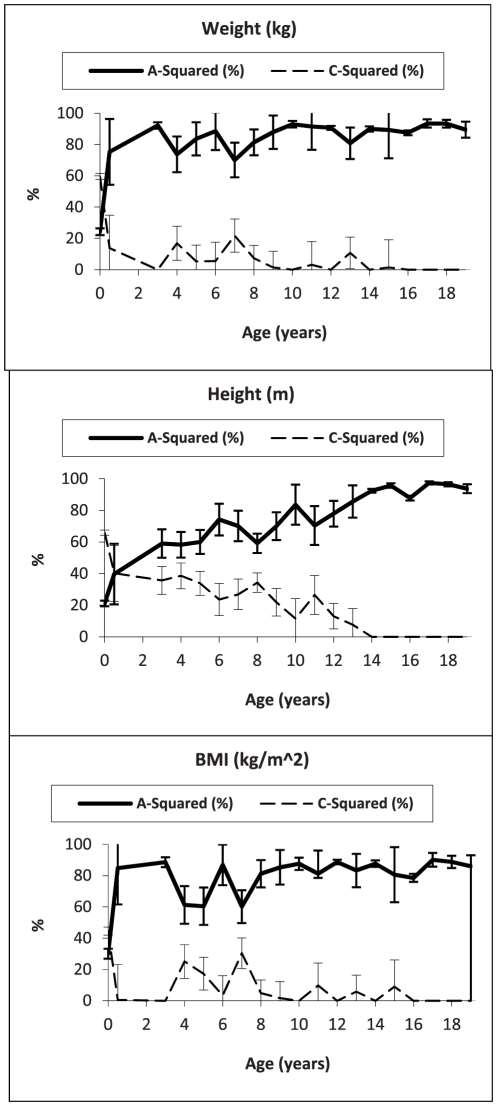
Proportion of the variance in weight (kg), height (m), and BMI (kg/m^2^) explained by A-squared and C-squared (with 95% confidence interval), in boys and girls (combined), from birth through 19 years of age – ACE models assumed.

The proportion of variance in weight, height, and BMI explained by additive genetic (a^2^), common environmental (c^2^), and unique environmental (e^2^) factors, according to full ACE and nested AE sex-limitation models from birth through 19 years of age, is presented for boys and girls in Tables 1 and 2, respectively; best fitting and most parsimonious models are displayed in bold in the tables. No sex-limitation was observed in either body weight or height at 4 and 7 years of age, nor in BMI at 9 years of age (data not shown); however, as significant sex-limitation was observed in all variables at every other age, all modeling results are presented in sex-limited form for consistency in Tables 1 and 2. The proportion of the phenotypic variance in weight, height and BMI explained by a^2^ and c^2^ according to the full ACE non-sex-limited model (with 95% confidence intervals), from birth through 19 years, are presented in Figure 4.

**Table 1:** Best fitting model (in bold) for weight, height, and BMI, from birth through age 19 years, and the proportion of variance explained by additive genetic (a^2^), common environmental (c^2^), and unique environmental (e^2^) influences: ACE-AE sex-limited model results for boys only.

	Weight					Height					BMI				
	ACE			AE		ACE			AE		ACE			AE	
Age *(Cohorts included)*	A^2^	C^2^	E^2^	A^2^	E^2^	A^2^	C^2^	E^2^	A^2^	E^2^	A^2^	C^2^	E^2^	A^2^	E^2^
**Birth** *(All cohorts)*	**8.7**	**81.2**	**10.1**	83.9	16.1	**6.4**	**85.9**	**7.81**	86.7	13.3	**8.2**	**74.1**	**17.6**	77.8	22.2
**5 mos** *(QNTS)*	58.2	29.8	11.9	**86.3**	**13.7**	**18.5**	**65.4**	**16.2**	78.0	22.0	65.2	20.4	14.4	**85.8**	**14.2**
**3 y** *(DTR)*	47.3	45.7	7.0	**92.6**	**7.4**	**40.6**	**57.2**	**2.2**	96.7	3.3	41.3	48.9	9.8	**90.9**	**9.1**
**4 y** *(DTR)*	**60.5**	**31.4**	**8.1**	90.8	9.2	**38.7**	**58.4**	**2.9**	95.7	4.3	**47.9**	**42.2**	**9.9**	88.1	11.9
**5 y** *(DTR & QNTS))*	82.7	9.2	8.0	**91.6**	**8.4**	**42.5**	**52.5**	**4.0**	94.3	5.7	**64.5**	**24.6**	**10.9**	87.5	12.5
**6 y** *(DTR)*	62.6	31.1	6.3	**93.2**	**6.8**	**55.9**	**42.4**	**1.8**	97.6	2.4	70.6	19.1	10.3	**89.3**	**10.7**
**7 y** *(DTR)*	**58.2**	**33.9**	**7.9**	92.1	7.9	**51.7**	**45.9**	**2.4**	96.9	3.1	48.5	43.0	8.5	90.8	9.2
**8 y** (DTR, QNTS, & TCHAD)	**76.7**	**11.9**	**11.4**	88.8	11.2	**43.1**	**51.6**	**5.3**	92.7	7.3	**75.9**	**9.6**	**14.6**	86.5	13.5
**9 y** *(CATSS & DTR)*	78.7	10.3	11.0	**89.6**	**10.4**	**51.9**	**40.6**	**7.5**	90.9	9.1	78.6	9.1	12.3	**87.6**	**12.4**
**10 y** *(DTR)*	92.2	0.0	7.8	**92.2**	**7.8**	**66.7**	**29.6**	**3.6**	95.7	4.3	87.0	0.0	13.0	**87.3**	**12.7**
**11 y** *(DTR)*	93.9	0.8	5.3	**95.5**	**4.5**	**60.4**	**38.0**	**1.6**	98.2	1.8	73.0	20.6	6.4	**94.2**	**5.8**
**12 y** (CATSS, DTR, & BTLS)	88.7	1.1	10.2	**90.2**	**9.8**	**68.1**	**23.0**	**9.0**	90.0	10.0	86.0	2.0	11.9	**88.1**	**11.9**
**13 y** *(DTR &TCHAD)*	**56.0**	**37.0**	**7.0**	92.0	8.0	63.5	30.6	5.9	**93.0**	**7.0**	70.3	19.7	9.9	**89.6**	**10.4**
**14 y** *(DTR & BTLS)*	88.8	0.0	11.2	**89.0**	**11.0**	77.7	15.5	6.9	**92.4**	**7.6**	86.0	0.9	13.1	**88.2**	**11.8**
**15 y** *(DTR)*	67.2	25.5	7.4	**92.2**	**7.8**	87.1	7.8	5.2	**94.8**	**5.2**	76.9	14.4	8.7	**90.5**	**9.5**
**16 y** *(DTR, BTLS, & TCHAD)*	**67.8**	**15.9**	**16.3**	84.2	15.8	**71.8**	**9.4**	**18.8**	80.2	19.8	**73.6**	**0.0**	**26.4**	73.7	26.3
**17 y** *(DTR)*	92.0	0.0	8.0	**92.0**	**8.0**	72.7	21.9	5.4	**93.9**	**6.1**	90.6	0.0	9.4	**91.0**	**9.0**
**18 y** *(DTR)*	90.8	3.3	5.9	**94.2**	**5.8**	79.8	8.9	11.3	**90.8**	**9.2**	86.6	1.1	12.3	**91.4**	**8.6**
**19 y** *(DTR)*	82.8	0.0	17.2	**84.3**	**15.7**	71.8	12.5	15.7	**82.9**	**17.1**	89.1	0.0	10.9	**90.1**	**9.9**

*Note: CE sex-limited model excluded as it never provided the best fit.*

**Table 2:** Best fitting model (in bold) for weight, height, and BMI, from birth through age 19 years, and the proportion of variance explained by additive genetic (a^2^), common environmental (c^2^), and unique environmental (e^2^) influences: ACE-AE sex-limited model results for girls only.

	Weight					Height					BMI				
	ACE			AE		ACE			AE		ACE			AE	
Age *(Cohorts)*	A^2^	C^2^	E^2^	A^2^	E^2^	A^2^	C^2^	E^2^	A^2^	E^2^	A^2^	C^2^	E^2^	A^2^	E^2^
**Birth** *(All cohorts)*	**4.9**	**84.9**	**10.2**	85.0	15.0	**4.8**	**87.3**	**7.91**	87.4	12.6	**7.9**	**74.2**	**17.8**	76.5	23.5
**5 mos** *(QNTS)*	70.9	19.5	9.7	**89.5**	**10.5**	**18.1**	**68.2**	**13.7**	80.8	19.2	76.9	7.0	16.2	**83.7**	**16.3**
**3 y** *(DTR)*	54.4	38.0	7.6	**91.6**	**8.4**	**31.0**	**64.0**	**5.0**	93.0	7.0	48.0	39.8	12.1	**86.7**	**13.3**
**4 y** *(DTR)*	**59.4**	**32.7**	**7.9**	90.8	9.2	**40.2**	**58.2**	**1.7**	97.5	2.5	**45.9**	**41.6**	**12.5**	86..1	13.9
**5 y** *(DTR & QNTS))*	78.6	8.3	13.1	**86.4**	**13.6**	**43.3**	**51.4**	**5.2**	93.1	6.9	**50.6**	**25.0**	**24.3**	73.8	26.2
**6 y** *(DTR)*	47.6	48.5	3.8	**95.4**	**4.6**	**54.5**	**43.7**	**1.8**	97.7	2.3	29.6	64.0	6.4	**91.9**	**8.1**
**7 y** *(DTR)*	**48.2**	**46.0**	**5.8**	91.8	8.2	**50.2**	**47.4**	**2.4**	96.8	3.2	**40.7**	**53.3**	**5.9**	91.6	8.4
**8 y** (DTR, QNTS, & TCHAD)	**66.6**	**23.7**	**9.6**	88.6	11.4	**42.9**	**52.7**	**4.4**	93.8	6.2	**61.8**	**26.4**	**11.8**	85.6	14.4
**9 y** *(CATSS & DTR)*	73.1	16.2	10.7	**88.8**	**11.2**	**49.0**	**45.2**	**5.8**	92.9	7.1	76.1	10.7	13.2	**86.4**	**13.6**
**10 y** *(DTR)*	94.0	0.0	6.0	**94.0**	**6.0**	**76.8**	**17.8**	**5.4**	94.1	5.9	81.5	7.1	11.4	**88.5**	**11.5**
**11 y** *(DTR)*	71.6	22.8	5.6	**93.5**	**6.5**	**56.5**	**50.4**	**3.1**	95.4	4.6	52.7	36.0	11.4	**86.3**	**13.7**
**12 y** (CATSS, DTR, & BTLS)	83.8	7.7	8.4	**91.1**	**8.9**	**62.5**	**30.4**	**7.1**	91.6	8.4	85.2	4.3	10.5	**89.2**	**10.8**
**13 y** *(DTR &TCHAD)*	**60.3**	**31.6**	**8.1**	90.6	9.4	63.9	30.3	5.8	**92.9**	**7.1**	65.5	23.8	10.6	**88.5**	**11.5**
**14 y** *(DTR & BTLS)*	88.0	2.8	9.2	**90.8**	**9.2**	56.8	35.7	7.5	**91.3**	**8.7**	73.1	15.2	11.7	**86.9**	**13.1**
**15 y** *(DTR)*	49.4	39.6	11.0	**87.9**	**12.1**	92.8	1.1	6.2	**93.8**	**6.2**	42.0	48.0	10.0	**88.8**	**11.2**
**16 y** *(DTR, BTLS, & TCHAD)*	**87.8**	**0.0**	**12.2**	87.7	12.3	**65.2**	**17.1**	**17.7**	81.0	19.0	**82.9**	**1.0**	**16.2**	83.9	16.1
**17 y** *(DTR)*	84.2	6.4	9.5	**90.6**	**9.4**	72.7	23.1	4.3	**95.2**	**4.8**	73.8	15.1	11.1	**89.4**	**10.6**
**18 y** *(DTR)*	39.8	49.6	10.6	**88.4**	**11.6**	96.2	0.0	3.8	**96.5**	**3.5**	51.6	37.7	10.7	**86.1**	**13.9**
**19 y** *(DTR)*	73.9	12.1	14.0	**85.9**	**14.1**	60.4	32.8	6.8	**92.4**	**7.6**	65.1	17.8	17.1	**80.7**	**19.3**

*Note: CE sex-limited model excluded as it never provided the best fit.*

In both sexes, weight and BMI shared similar aetiologies; however, the aetiology of body height differed from other body measures. ACE was the best-fitting model for weight only at birth and at ages 4, 7, 8, 13, and 16 years. Similarly, for BMI, ACE was the best-fitting model at birth and at ages 4, 5, 7, 8, and 16 years. For all other ages an AE model provided best fit for weight and BMI (Tables 1 and 2). With regard to height, the effect of the common environment played a more important role, with the ACE model being consistently chosen as the best-fitting model from birth through 12 years of age, and again at 16 years of age. Only from 13 through 15 years of age, and from 17 through 19 years was AE the best-fitting model for height.

**Figure 5:**
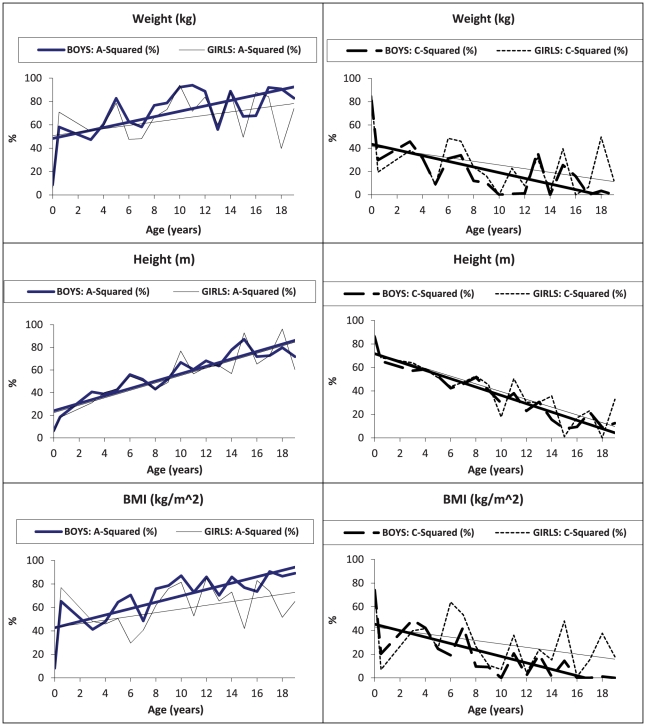
Proportion of the variance (with linear trend) in weight (kg), height (m), and BMI (kg/m^2^) explained by A-squared and C-squared, in boys and girls separately, from birth through 19 years of age – ACE models assumed.

Figure 5 presents the proportion of phenotypic variance in weight, height, and BMI explained by a^2^ and c^2^ (according to the full ACE sex-limitation model) from birth through 19 years of age, in boys and girls separately. The proportion of variance in body weight and BMI explained by genetic influence was greater in boys than in girls, with the gap between the sexes increasing consistently from 4 through 19 years of age. This sex difference was significant, but less apparent for height. For girls, the effect of the common environment played a more important role, particularly in explaining the variability in BMI. For both sexes, heritability in variances for body weight, height, and BMI was low at birth, between 6.4 and 8.7% in all measures for boys and between 4.8 and 7.9% in all measures for girls, but increased over time. Genetic effects accounted for close to half or more of the variance in weight and BMI after 5 months of age in both sexes, while the effect of the common environment in all body measures was high at birth, between 74.1 and 85.9% in all measures for boys and between 74.2 and 87.3% in all measures for girls, and markedly reduced over time. For body height, however, the effect of the common environment maintained a greater influence over a longer period during early childhood (from birth up to 12 years of age), in comparison to its influence on body weight or BMI. The effect of the unique environment generally remained stable for both sexes, across all body measures, and at all ages, accounting for less than 19% (ACE models) of the variance in weight, height, and BMI from birth through age 19 years, with the exception for BMI at age 16 years in boys (26.4%) and at 5 years in girls (24.3%), where the effect was slightly higher.

## Discussion

While others have conducted international twin studies to compare the heritability of body height and/or BMI during certain years in adolescence [44] and over broader age ranges in adulthood [9], [45], the present study is unique for its description and examination of the genetic and environmental influences on body weight, height, and BMI over virtually every year of age, from birth through 19 years, in a large sample of MZ and DZ same- and opposite-sex twins from three continents. The findings indicate that variability in weight, height, and BMI amongst twins from four developed countries is strongly influenced by genetic factors in both sexes as early as from 5 months of age, and increasingly so through late adolescence. The increasing heritability in these body measures over time, which was found to explain at times more than 80–90% of the variance in mid- to late adolescence, was observed along with a decreased influence of the common environment in early childhood years. Common environmental influences were found to play an important role in influencing variability in body weight, height, and BMI in early childhood in both sexes, particularly for body height where the influence of the common environment remained significant for a longer period, up through 12 years of age; however, common environmental influences were, for the most part, no longer significant by early- to mid-adolescence. On the other hand, the influence of the unique environment on all body measures was found to be small, but significant, at all ages and in both sexes.

### Increasing Heritability

The increasing heritability observed in the present study is in agreement with results from other large twin-studies which found similar results for BMI in twins followed longitudinally from 4 to 7 years of age [46] and from 11–12 to 14 and 17 years of age [19]. A large longitudinal study of Dutch twin children followed from ages 3 to 12 years also found strong evidence for the role of genetic influences on body height and BMI in males and females, in addition to moderate non-significant increases in heritability specifically for height [47]. In correspondence with the present study's findings, these increasing heritability estimates for height resulted from the decreased influence of the common environment; however, common environmental influences remained important for both height and BMI at all ages examined [47].

Increasing heritability estimates have also been observed in other traits over childhood, such as in cognitive development [48], and this is generally attributed in part to changing gene expression, but also to gene-environment interaction (G×E) [19], [49] and gene-environment correlations [50]–[52]. In the case of gene-environment interactions, individuals with differing genotypes may react to specific environmental stimuli in different ways. If a gene interacts with a factor within the common/shared environment, but the phenotypic expression associated with this interaction is only activated in adolescent years, this may explain the observed diminished effect of the common environment in early- to mid-adolescence in the present study. Alternatively, with gene-environment correlations, a genetic factor may influence an environmental exposure such that individuals may seek environments that correlate with the same phenotype. For example, an individual with a genetic predisposition for weight gain who is inclined to maintain a sedentary lifestyle or poor dietary practices may seek the company of other individuals who share similar attributes and practices, possibly increasing exposure to obesogenic environments that would lead to furthered weight gain. Such gene-environment correlations could increase heritability over time, firstly, due to increased independence to choose one's personal environment in adolescence, with MZ twins choosing more similar environments in comparison to DZ twins due to their identical genes, and, secondly, due to the genetic influences becoming more reinforced and direct over time by means of the respective correlated and phenotype-proliferating environmental influences. In the present study, the genetic modeling technique used includes “gene-environment interaction” within the component of heritability if the environmental component of the interaction is shared within a twin pair, and within the unique environment component if not shared [49]. Although effects of interaction and genotype-environment correlation were not quantified in the present study, these are not sufficient to fully explain the dramatic decrease in the independent effect of the common environment by early adolescence.

### Decreasing effect of the Common Environment

The present study's finding that the effect of the common environment was highest at birth, with heritability thus being the lowest at that point, is consistent with results from other twin studies [14], [47], [53]–[55]. This observation reiterates the special intrauterine situation characterized by twinning whereby, due to shared placental membranes and environmental/nutritional constraints in the uterus, monochorionic MZ twins may compete more intensely for prenatal resources than dichorionic DZ twins, making them less similar at birth than their genetic potential would allow [22], [56]. However, the influence of the common environment decreased rapidly through later childhood years, particularly for body weight and BMI. In correspondence, a longitudinal study of Finnish twins found persistent effects of the common environment on variations in BMI up through 14 years of age, but a disappearance of this influence by 17 years of age [19]. A recent systematic review on twin and adoption studies also reported a substantial effect of the common environment on variations in BMI in mid-childhood, but a disappearance of this effect in adolescence [10]. Several studies have reported significant effects of the common environment on BMI in children aged 12 years or less, but not over the age of 12 years [13], [47], [57], [58]. However, not all studies support this finding [54].

Our results suggest that potential common environmental factors exert their strongest and most independent influence on variations in weight, height, and BMI specifically in pre-adolescent years. However, this does not signify that environmental factors are irrelevant as targets for intervention once a child reaches adolescence; rather, this may signify a lack of common environmental influences that are independent of genetic predisposition in later adolescent years [14]. Furthermore, given the situation of gene-environment correlation and gene-environment interaction, it is all the more necessary to continue investigating potential environmental interventions that counter the obesity epidemic in those most genetically predisposed, as early in life as possible.

### Presence of Sex-limitation

The sex-difference observed in the present study revealed that the proportion of the variance explained by genetic influence was greater in boys than in girls over the majority of years examined; this was most pronounced for weight and BMI. This sex-difference appears to have resulted from a difference in the magnitude of the influence of the common environment across the sexes, which seems to have played a more important role in girls than in boys. The influence of the unique environment on all body measures was similar in both sexes across the ages examined. These sex-differences concur with findings from a longitudinal study of Finnish twins, which found that boys had slightly higher heritability estimates at all ages examined in comparison to girls [19], and studies that report higher heritability estimates for BMI in men than in women [8], [9]. However, some studies report higher heritability estimates in girls in comparison to boys [16]–[18].

The reasons behind these sex-differences are intriguing. From early infancy and through pre-puberty, sex-differences are observed not only in fat mass and pattern of fat distribution [59], [60], but also in hormone levels that are implicated in feeding behaviours, metabolic processes and body composition, e.g. insulin & leptin [61]–[63]. However, given the present study's findings, it is important to consider what common environmental factors may influence variances in body weight and BMI in girls, more so than boys. Some studies suggest sex-differences in childhood obesity, demonstrating that boys and girls differ in their susceptibility to various social and ethnic environmental influences [64]. Furthermore, environmental influences, such as the availability of unhealthy foods in the home or exposure to family conflicts, are seen to associate with obesity-promoting dietary practices, such as the consumption of sweet snacks or take-away, in girls more so than in boys [65]. Similarly, a meta-analysis found that in girls, but not in boys, increasing parental food restriction (i.e. the degree to which parents attempt to restrict their child's eating during meals) was associated with an increased tendency to eat in the absence of hunger [66]. An association between parental level of education and BMI has also been observed in adolescent females, but not in males [67]. Another study showed an association between girls' weight concerns and mother's gender attitudes, with no such association observed in boys [68]. Research is needed to examine potential sex-differences in gene-environment interaction that may occur as a result of underlying sex-differences in hormonal regulation (e.g. leptin) and its interaction with the family and social environmental influences mentioned above [64].

Finally, epigenetic factors relating to body weight, height, and BMI, must also be considered. The epigenetic model of obesity theorizes that maternal weight gain/obesity, and/or nutrition prior to, and during pregnancy may create permanent changes in the gene regulation of the foetus, promoting an obese phenotype [69]. Much remains to be understood about the role of epigenetics and how its effects may differ across the sexes. However, the twin modeling techniques used model epigenetic effects as part of the variation accounted for by the unique environment, and no notable differences were observed across the sexes for this component.

### Strengths & Limitations

The present study has important strengths. Firstly, it analyzes a large sample of MZ and DZ same- and opposite-sex twins from birth cohorts obtained from four different countries in three continents; this not only provides high power and high confidence in the study results, but also allows for a strong assessment of sex-limitation. This study also provides a comprehensive overview of the genetic and environmental influences on body weight, height, and BMI over the entire span of childhood and adolescence, with data available from birth, at 5 months of age, and yearly from 3 years to 19 years of age, inclusively.

A limitation of this study lies in the use of self- or parent-reported weights and heights for some cohorts, which may have caused some bias due to the common under-reporting of weight known to occur especially in individuals with true values in the upper end of the BMI distribution [70], [71]. However, there is no reason to suspect a difference in the degree of bias across a twin pair, with an exception perhaps for dizygotic twins of the opposite sex, since girls have been shown to under-report their weight to a much larger extent in comparison to boys [72]. It is also reported that mothers overestimate their children's weights more than their heights, especially for boys, such that this was found to overestimate the prevalence of overweight by over 3% in 4-year-old children, and by 5% specifically for boys [14]. However, another study reports that parents generally tend to underestimate the prevalence of overweight in young children, typically by underreporting body weight and over-reporting height in children with a high BMI, and over-reporting body weight in children with a low BMI [73]. Such a potential bias may lead to decreased heritability estimates and increased estimates for the effect of the unique environment. A bias specifically across the sexes, though, would mainly affect the results obtained through the sex-limitation analyses, leaving the general analyses unaffected. Nonetheless, it is important to note that, several twin studies from various populations (Australian, Finnish, Danish, and British) have reported a good agreement between self-reported and measured weight and height [9], [74].

Due to the lack of information about the weight, height and BMI of parents, another limitation arises with the techniques used in twin analyses. Without such information, one must make the assumption of random mating. Several studies, however, have shown that this is an unrealistic assumption as assortative mating, the increased preference to marry someone with similar traits, occurs with several phenotypic, physical, and psychological characteristics, including BMI [75], [76], [76], [77]. Assortative mating may potentially inflate heritability estimates in twin studies since it increases the genetic similarities between DZ twins above the 0.5 correlation assumed in the twin modeling techniques. Furthermore, even if twin modeling techniques reveal that an AE model best fits the data (i.e. no effect of the common environment observed), this does not mean that assortative mating and non-additivity are not acting [78]. On the other hand, assortative mating may also inflate the variance estimates obtained as part of the common environmental component when not accounted for in the modeling, since this estimate is derived from comparing correlations between MZ and DZ twin pairs with an assumption of a correlation of 0.5 between DZ twin pairs. However, given that the effect of the common environment became insignificant after early adolescence, it is very unlikely that assortative mating would have significantly inflated the estimates obtained for that component. We are thus confident that the effect of the common environment observed in early childhood years is in fact a true influence and is not simply an artificial result of the twin-modeling techniques used [10]. Finally, the present study's findings may be limited in their generalizability to other ethnicities due to the main inclusion of Caucasian populations.

### Conclusions and Future Directions

Genetics appear to play an increasingly important role in explaining the variation in weight, height, and BMI from early childhood to late adolescence, with boys being more significantly affected by these effects. This finding emphasizes the need for future studies to continue identifying common genetic variants that may predispose individuals to obesity. It is hoped that with the identification of such variants and a furthered understanding of potential gene-environment interactions, interventions may be tailored to an individual's personal genetic predisposition so that greater success can be attained in the battle against obesity. The findings also emphasize the need to target family and social environmental interventions in early childhood years, particularly for females, as the effect of the common environment was particularly influential until early adolescence.

## Supporting Information

Table S1 **Sample sizes and characteristics of the cohorts included in the analyses.**
(PDF)

Table S2 **Mean and Standard Error of weight (kg) in MZ and DZ twins of four countries, from birth through 19 years of age.**
(PDF)

Table S3 **Mean and Standard Error of height (m) in MZ and DZ twins of four countries, from birth through 19 years of age.**
(PDF)

Table S4 **Mean and Standard Error of BMI (kg/m^2^) in MZ and DZ twins of four countries, from birth through 19 years of age.**
(PDF)

Table S5 **Intra-class correlations (ICC) between MZ and DZ twin pairs for mean weight (kg), height (m), and BMI (kg/m^2^), from birth through 19 years of age.**
(PDF)
