# Supplementary material for: Genetic and Environmental Contributions to Weight, Height, and BMI from Birth to 19 Years of Age: An International Study of Over 12,000 Twin Pairs
Source: PLoS One. 2012 Feb 8;7(2):e30153. doi: 10.1371/journal.pone.0030153 (PMC3275599; doi:10.1371/journal.pone.0030153)
Supplement: Table S1 — Sample sizes and characteristics of the cohorts included in the analyses. (PDF) [file pone.0030153.s001.pdf]

Table S1: Sample sizes and characteristics of the cohorts included in the analyses

Birth Cohort	Age of data collection	N of MZ male twin pairs included	N of MZ female twin pairs included	N MZ Total Same-sex twin pairs included	N DZ Same-sex male twin pairs included	N DZ Same-sex female twin pairs included	N DZ Total Same-sex twin pairs included	N DZ Opposite-sex twin pairs included	N of twin pairs included
QNTS	Birth	80	86	166	60	54	111	111	388
	5mos	55	68	123	51	48	99	81	303
	5y	80	86	166	60	51	111	111	388
	8y	67	78	145	55	54	109	107	361
CATSS	Birth	91	81	172	96	71	167	173	512
	9y	80	73	153	92	70	162	173	488
	12y	91	81	172	96	71	167	163	502
TCHAD	Birth	186	190	376	131	138	169	278	823
	8y	186	182	368	126	123	249	278	895
	13y	158	160	318	108	119	227	235	780
	16y	185	190	375	131	138	269	275	919
DTR	Birth	76	65	141	183	171	352	300	793
	3y	53	65	118	170	156	326	300	744
	4y	57	64	121	181	171	352	286	759
	5y	69	56	125	183	151	334	288	747
	6y	76	57	133	165	139	304	254	691
	7y	59	60	119	152	148	300	288	707
	8y	44	54	98	157	128	285	238	621
	9y	55	49	104	136	137	273	249	626
	10y	60	45	105	121	139	260	212	577
	11y	44	54	98	117	111	228	170	496
	12y	39	44	83	79	100	179	165	427
	13y	39	32	71	83	93	176	119	366
	14y	41	37	78	75	82	157	112	347
	15y	43	41	84	93	87	180	114	378
	16y	38	42	80	61	56	117	94	291
	17y	31	31	62	62	72	134	72	268
	18y	36	37	73	45	46	91	65	229
	19y	24	26	50	36	44	80	74	204
MOLE	Birth	132	130	262	139	126	265	227	754
	12y	130	130	260	139	126	265	227	752
	14y	132	139	271	121	120	241	200	712
MAPS	Birth	116	123	239	85	82	167	170	576
	16y	116	123	239	85	82	167	170	576
