# Supplementary material for: Genetic and Environmental Contributions to Weight, Height, and BMI from Birth to 19 Years of Age: An International Study of Over 12,000 Twin Pairs
Source: PLoS One. 2012 Feb 8;7(2):e30153. doi: 10.1371/journal.pone.0030153 (PMC3275599; doi:10.1371/journal.pone.0030153)
Supplement: Table S2 — Mean and Standard Error of weight (kg) in MZ and DZ twins of four countries, from birth through 19 years of age. (PDF) [file pone.0030153.s002.pdf]

Table S2: Mean and Standard Error of weight (kg) in MZ and DZ twins of four countries, from birth through 19 years of age.

	MZ Twins									DZ Twins												All Twins		
	Boys			Girls			Total			Boys			Girls			Opposite-sex			Total			Total		
Age *(Cohorts)*	N	Mean	*SE*	N	Mean	*SE*	N	Mean	*SE*	N	Mean	*SE*	N	Mean	*SE*	N	Mean	*SE*	N	Mean	*SE*	N	Mean	*SE*
Birth *(All cohorts)*	3159	2.6	0.01	3126	2.5	0.01	6285	2.5	0.01	4788	2.6	0.01	4578	2.6	0.01	8385	2.6	0.01	17751	2.6	0.00	24036	2.6	0.00
5 mos *(QNTS)*	110	7.8	0.09	136	7.1	0.08	246	7.4	0.06	102	7.7	0.09	96	7.1	0.09	162	7.4	0.07	360	7.4	0.05	606	7.4	0.04
3y *(DTR)*	106	15.1	0.19	130	15.2	0.17	236	15.2	0.13	340	15.2	0.11	312	14.5	0.11	600	14.8	0.08	1252	14.8	0.06	1488	14.9	0.05
4y *(DTR)*	114	17.2	0.21	128	17.0	0.20	242	17.1	0.14	362	17.2	0.12	342	16.9	0.12	572	17.0	0.09	1276	17.0	0.06	1518	17.0	0.06
5y *(DTR & QNTS))*	298	19.0	0.16	284	18.8	0.16	582	18.9	0.12	486	19.8	0.13	404	19.0	0.14	798	19.1	0.10	1688	19.3	0.07	2270	19.2	0.06
6y *(DTR)*	152	21.8	0.26	114	21.3	0.30	266	21.6	0.20	330	21.9	0.18	278	21.6	0.19	508	21.8	0.14	1116	21.8	0.10	1382	21.8	0.09
7y *(DTR)*	118	24.2	0.36	120	24.4	0.36	238	24.3	0.26	304	24.3	0.23	296	24.4	0.23	576	24.3	0.16	1176	24.3	0.12	1414	24.3	0.10
8y (DTR, QNTS, & TCHAD)	594	28.9	0.22	628	28.2	0.21	1222	28.5	0.15	676	28.8	0.20	610	28.7	0.22	1246	28.5	0.15	2532	28.6	0.11	3754	28.6	0.09
9y *(CATSS & DTR)*	270	30.8	0.34	244	30.0	0.35	514	30.4	0.24	456	31.4	0.26	414	31.0	0.27	844	31.0	0.19	1714	31.1	0.13	2228	30.9	0.12
10y *(DTR)*	120	33.7	0.58	90	33.5	0.68	210	33.6	0.44	242	35.9	0.41	278	34.0	0.38	424	34.5	0.31	944	34.7	0.21	1154	34.5	0.19
11y *(DTR)*	88	37.1	0.74	108	37.3	0.67	196	37.2	0.50	234	38.2	0.46	222	38.3	0.47	340	37.8	0.38	796	38.1	0.25	992	37.9	0.22
12y (CATSS, DTR, & BTLS)	520	41.7	0.40	510	43.5	0.40	1030	42.6	0.28	628	42.8	0.36	594	43.5	0.37	1110	42.3	0.27	2332	42.7	0.19	3362	42.7	0.16
13y *(DTR &TCHAD)*	394	49.7	0.46	384	49.1	0.46	778	49.4	0.33	382	51.7	0.47	424	48.8	0.44	708	49.9	0.34	1514	50.1	0.24	2292	49.8	0.19
14y *(DTR & BTLS)*	346	53.9	0.58	352	52.5	0.57	698	53.1	0.41	392	55.3	0.54	404	53.0	0.54	624	54.9	0.43	1420	54.5	0.29	2118	54.0	0.23
15y *(DTR)*	86	58.5	1.03	82	55.0	1.06	168	56.8	0.75	186	60.2	0.70	174	56.0	0.72	228	59.6	0.63	588	58.7	0.40	756	58.3	0.35
16y *(DTR, BTLS, & TCHAD)*	678	65.3	0.42	710	56.3	0.41	1388	60.7	0.31	554	66.7	0.46	552	58.3	0.46	1078	62.7	0.33	2184	62.6	0.25	3572	61.9	0.19
17y *(DTR)*	62	69.9	1.38	62	58.0	1.38	124	64.0	1.07	124	70.5	0.98	144	59.1	0.91	144	64.7	0.91	412	64.5	0.59	536	64.4	0.51
18y *(DTR)*	72	69.2	1.44	74	63.0	1.42	146	66.1	1.09	90	73.9	1.29	92	59.3	1.28	130	67.3	1.07	312	66.9	0.75	458	66.6	0.61
19y *(DTR)*	48	74.8	1.57	52	60.8	1.50	100	67.5	1.23	72	75.8	1.28	88	60.8	1.16	148	68.9	0.89	308	68.2	0.70	408	68.0	0.61
