# Supplementary material for: Genetic and Environmental Contributions to Weight, Height, and BMI from Birth to 19 Years of Age: An International Study of Over 12,000 Twin Pairs
Source: PLoS One. 2012 Feb 8;7(2):e30153. doi: 10.1371/journal.pone.0030153 (PMC3275599; doi:10.1371/journal.pone.0030153)
Supplement: Table S3 — Mean and Standard Error of height (m) in MZ and DZ twins of four countries, from birth through 19 years of age. (PDF) [file pone.0030153.s003.pdf]

Table S3: Mean and Standard Error of height (m) in MZ and DZ twins of four countries, from birth through 19 years of age

	MZ Twins									DZ Twins												All Twins		
	Boys			Girls			Total			Boys			Girls			Opposite-sex			Total			Total		
Age *(Cohorts)*	N	Mean	*SE*	N	Mean	*SE*	N	Mean	*SE*	N	Mean	*SE*	N	Mean	*SE*	N	Mean	*SE*	N	Mean	*SE*	N	Mean	*SE*
Birth *(All cohorts)*	3159	0.47	0.001	3126	0.47	0.001	6285	0.47	0.000	4788	0.48	0.000	4578	0.48	0.000	8385	0.48	0.000	17751	0.48	0.000	24036	0.48	0.000
5 mos *(QNTS)*	110	0.65	0.002	136	0.63	0.002	246	0.64	0.002	102	0.65	0.002	96	0.63	0.003	162	0.64	0.002	360	0.64	0.001	606	0.64	0.001
3y *(DTR)*	106	0.98	0.004	130	0.98	0.004	236	0.98	0.003	340	0.98	0.002	312	0.96	0.003	600	0.97	0.002	1252	0.97	0.001	1488	0.97	0.001
4y *(DTR)*	114	1.05	0.005	128	1.05	0.004	242	1.05	0.003	362	1.05	0.003	342	1.05	0.003	572	1.05	0.002	1276	1.05	0.001	1518	1.05	0.001
5y *(DTR & QNTS))*	298	1.10	0.003	284	1.10	0.003	582	1.10	0.002	486	1.12	0.002	404	1.11	0.003	798	1.12	0.002	1688	1.12	0.001	2270	1.11	0.001
6y *(DTR)*	152	1.19	0.005	114	1.18	0.005	266	1.18	0.003	330	1.19	0.003	278	1.18	0.003	508	1.19	0.002	1116	1.19	0.002	1382	1.19	0.002
7y *(DTR)*	118	1.25	0.006	120	1.24	0.006	238	1.24	0.004	304	1.25	0.003	296	1.25	0.004	576	1.25	0.003	1176	1.25	0.002	1414	1.25	0.002
8y (DTR, QNTS, & TCHAD)	594	1.33	0.003	628	1.32	0.003	1222	1.32	0.002	676	1.33	0.002	610	1.32	0.003	1246	1.32	0.002	2532	1.32	0.001	3754	1.32	0.001
9y *(CATSS & DTR)*	270	1.36	0.004	244	1.35	0.004	514	1.35	0.003	456	1.38	0.003	414	1.36	0.003	844	1.36	0.002	1714	1.37	0.002	2228	1.36	0.001
10y *(DTR)*	120	1.41	0.006	90	1.41	0.007	210	1.41	0.005	242	1.44	0.004	278	1.41	0.004	424	1.43	0.003	944	1.42	0.002	1154	1.42	0.002
11y *(DTR)*	88	1.49	0.008	108	1.47	0.008	196	1.48	0.006	234	1.49	0.005	222	1.48	0.005	340	1.48	0.004	796	1.48	0.003	992	1.48	0.003
12y (CATSS, DTR, & BTLS)	520	1.51	0.003	510	1.52	0.003	1030	1.52	0.002	628	1.52	0.003	594	1.53	0.003	1110	1.52	0.002	2332	1.52	0.002	3362	1.52	0.001
13y *(DTR &TCHAD)*	394	1.63	0.004	384	1.61	0.004	778	1.62	0.003	382	1.63	0.004	424	1.61	0.004	708	1.61	0.003	1514	1.62	0.002	2292	1.62	0.002
14y *(DTR & BTLS)*	346	1.66	0.004	352	1.61	0.004	698	1.63	0.003	392	1.66	0.004	404	1.63	0.004	624	1.64	0.003	1420	1.64	0.002	2118	1.64	0.002
15y *(DTR)*	86	1.73	0.008	82	1.64	0.008	168	1.69	0.006	186	1.74	0.006	174	1.66	0.006	228	1.71	0.005	588	1.71	0.003	756	1.70	0.003
16y *(DTR, BTLS, & TCHAD)*	678	1.76	0.003	710	1.64	0.003	1388	1.70	0.002	554	1.77	0.003	552	1.66	0.003	1078	1.71	0.002	2184	1.71	0.002	3572	1.71	0.002
17y *(DTR)*	62	1.81	0.009	62	1.67	0.009	124	1.74	0.008	124	1.80	0.007	144	1.68	0.006	144	1.74	0.006	412	1.74	0.004	536	1.74	0.004
18y *(DTR)*	72	1.80	0.009	74	1.69	0.009	146	1.75	0.008	90	1.81	0.008	92	1.67	0.008	130	1.75	0.007	312	1.75	0.005	458	1.75	0.004
19y *(DTR)*	48	1.84	0.011	52	1.68	0.010	100	1.75	0.009	72	1.81	0.009	88	1.69	0.008	148	1.74	0.006	308	1.74	0.005	408	1.75	0.005
