# Supplementary material for: Genetic and Environmental Contributions to Weight, Height, and BMI from Birth to 19 Years of Age: An International Study of Over 12,000 Twin Pairs
Source: PLoS One. 2012 Feb 8;7(2):e30153. doi: 10.1371/journal.pone.0030153 (PMC3275599; doi:10.1371/journal.pone.0030153)
Supplement: Table S4 — Mean and Standard Error of BMI (kg/m2) in MZ and DZ twins of four countries, from birth through 19 years of age. (PDF) [file pone.0030153.s004.pdf]

Table S4: Mean and Standard Error of BMI (kg/m^2) in MZ and DZ twins of four countries, from birth through 19 years of age.

	MZ Twins									DZ Twins												All Twins		
	Boys			Girls			Total			Boys			Girls			Opposite-sex			Total			Total		
Age *(Cohorts)*	N	Mean	*SE*	N	Mean	*SE*	N	Mean	*SE*	N	Mean	*SE*	N	Mean	*SE*	N	Mean	*SE*	N	Mean	*SE*	N	Mean	*SE*
Birth *(All cohorts)*	3159	11.33	0.03	3126	11.27	0.03	6285	11.30	0.02	4788	11.32	0.02	4578	11.25	0.02	8385	11.39	0.02	17751	11.34	0.01	24036	11.33	0.01
5 mos *(QNTS)*	110	18.46	0.17	136	17.79	0.15	246	18.09	0.11	102	18.44	0.17	96	17.59	0.18	162	17.99	0.14	360	18.01	0.09	606	18.04	0.07
3y *(DTR)*	106	15.79	0.15	130	15.69	0.13	236	15.74	0.10	340	15.90	0.08	312	15.60	0.09	600	15.78	0.06	1252	15.77	0.04	1488	15.76	0.04
4y *(DTR)*	114	15.68	0.13	128	15.43	0.12	242	15.55	0.09	362	15.53	0.07	342	15.38	0.08	572	15.43	0.06	1276	15.45	0.04	1518	15.46	0.04
5y *(DTR & QNTS))*	298	15.50	0.09	284	15.39	0.09	582	15.45	0.06	486	15.62	0.07	404	15.32	0.08	798	15.35	0.06	1688	15.42	0.04	2270	15.43	0.03
6y *(DTR)*	152	15.37	0.13	114	15.31	0.15	266	15.34	0.10	330	15.33	0.09	278	15.41	0.09	508	15.41	0.07	1116	15.39	0.05	1382	15.38	0.04
7y *(DTR)*	118	15.51	0.18	120	15.75	0.17	238	15.63	0.12	304	15.51	0.11	296	15.65	0.11	576	15.45	0.08	1176	15.52	0.06	1414	15.54	0.05
8y (DTR, QNTS, & TCHAD)	594	16.31	0.09	628	16.19	0.09	1222	16.25	0.06	676	16.30	0.08	610	16.43	0.09	1246	16.31	0.06	2532	16.33	0.04	3754	16.31	0.04
9y *(CATSS & DTR)*	270	16.70	0.14	244	16.43	0.14	514	16.57	0.10	456	16.51	0.11	414	16.70	0.11	844	16.60	0.08	1714	16.60	0.05	2228	16.59	0.05
10y *(DTR)*	120	16.77	0.21	90	16.70	0.24	210	16.74	0.16	242	17.34	0.14	278	16.97	0.13	424	16.83	0.11	944	17.00	0.07	1154	16.95	0.07
11y *(DTR)*	88	16.74	0.25	108	17.09	0.23	196	16.93	0.17	234	17.25	0.15	222	17.39	0.16	340	17.21	0.13	796	17.27	0.08	992	17.20	0.07
12y (CATSS, DTR, & BTLS)	520	18.18	0.13	510	18.64	0.13	1030	18.41	0.09	628	18.47	0.12	594	18.43	0.12	1110	18.15	0.09	2332	18.31	0.06	3362	18.34	0.05
13y *(DTR &TCHAD)*	394	18.62	0.13	384	18.96	0.13	778	18.79	0.09	382	19.32	0.14	424	18.82	0.13	708	19.07	0.10	1514	19.06	0.07	2292	18.97	0.06
14y *(DTR & BTLS)*	346	19.53	0.17	352	20.21	0.17	698	19.87	0.12	392	19.85	0.16	404	19.86	0.16	624	20.33	0.13	1420	20.06	0.09	2118	20.00	0.07
15y *(DTR)*	86	19.45	0.31	82	20.39	0.32	168	19.91	0.22	186	19.90	0.21	174	20.28	0.22	228	20.21	0.19	588	20.13	0.12	756	20.08	0.10
16y *(DTR, BTLS, & TCHAD)*	678	21.13	0.13	710	20.79	0.13	1388	20.96	0.09	554	21.32	0.14	552	21.16	0.14	1078	21.44	0.10	2184	21.34	0.07	3572	21.19	0.06
17y *(DTR)*	62	21.30	0.37	62	20.71	0.37	124	21.00	0.27	124	21.84	0.26	144	21.01	0.25	144	21.23	0.25	412	21.34	0.15	536	21.26	0.13
18y *(DTR)*	72	21.28	0.40	74	22.05	0.39	146	21.67	0.28	90	22.43	0.36	92	21.18	0.35	130	21.71	0.30	312	21.76	0.19	458	21.73	0.16
19y *(DTR)*	48	22.14	0.42	52	21.53	0.41	100	21.83	0.30	72	23.05	0.35	88	21.24	0.31	148	22.58	0.24	308	22.31	0.17	408	22.19	0.15
