# Supplementary material for: Genetic and Environmental Contributions to Weight, Height, and BMI from Birth to 19 Years of Age: An International Study of Over 12,000 Twin Pairs
Source: PLoS One. 2012 Feb 8;7(2):e30153. doi: 10.1371/journal.pone.0030153 (PMC3275599; doi:10.1371/journal.pone.0030153)
Supplement: Table S5 — Intra-class correlations (ICC) between MZ and DZ twin pairs for mean weight (kg), height (m), and BMI (kg/m2), from birth through 19 years of age. (PDF) [file pone.0030153.s005.pdf]

Table S5: Intra-class correlations (ICC) between MZ and DZ twin pairs for mean weight (kg), height (m), and BMI (kg/m^2), from birth through 19 years of age.

ICC for Weight (kg)							
	MZ Twins			DZ Twins			
Age *(Cohorts)*	Boys	Girls	Total	Boys	Girls	Opposite-sex	Total
Birth *(All cohorts)*	0.833 [a]	0.821 [b]	0.828 [c]	0.725 [a]	0.752 [b]	0.695	0.719 [c]
5 mos *(QNTS)*	0.883 [a]	0.886 [b]	0.899 [c]	0.600 [a]	0.439 [b]	0.385	0.503 [c]
3y *(DTR)*	0.927 [a]	0.951 [b]	0.942 [c]	0.625 [a]	0.528 [b]	0.312	0.443 [c]
4y *(DTR)*	0.906 [a]	0.903 [b]	0.905 [c]	0.521 [a]	0.554 [b]	0.534	0.538 [c]
5y *(DTR & QNTS))*	0.927 [a]	0.860 [b]	0.896 [c]	0.441 [a]	0.413 [b]	0.491	0.465 [c]
6y *(DTR)*	0.910 [a]	0.941 [b]	0.923 [c]	0.506 [a]	0.680 [b]	0.434	0.508 [c]
7y *(DTR)*	0.886 [a]	0.931 [b]	0.913 [c]	0.556 [a]	0.655 [b]	0.523	0.570 [c]
8y (DTR, QNTS, & TCHAD)	0.860 [a]	0.901 [b]	0.883 [c]	0.519 [a]	0.459 [b]	0.482	0.484 [c]
9y *(CATSS & DTR)*	0.893 [a]	0.869 [b]	0.884 [c]	0.519 [a]	0.548 [b]	0.365	0.457 [c]
10y *(DTR)*	0.911 [a]	0.907 [b]	0.911 [c]	0.229 [a]	0.439 [b]	0.479	0.408 [c]
11y *(DTR)*	0.928 [a]	0.960 [b]	0.952 [c]	0.555 [a]	0.561 [b]	0.356	0.482 [c]
12y (CATSS, DTR, & BTLS)	0.889 [a]	0.917 [b]	0.905 [c]	0.362 [a]	0.490 [b]	0.427	0.427 [c]
13y *(DTR &TCHAD)*	0.914 [a]	0.909 [b]	0.912 [c]	0.599 [a]	0.554 [b]	0.433	0.515 [c]
14y *(DTR & BTLS)*	0.865 [a]	0.925 [b]	0.899 [c]	0.380 [a]	0.368 [b]	0.347	0.368 [c]
15y *(DTR)*	0.905 [a]	0.897 [b]	0.904 [c]	0.551 [a]	0.606 [b]	0.237	0.460 [c]
16y *(DTR, BTLS, & TCHAD)*	0.837 [a]	0.871 [b]	0.874 [c]	0.490 [a]	0.383 [b]	0.164	0.340 [c]
17y *(DTR)*	0.919 [a]	0.949 [b]	0.948 [c]	0.126 [a]	0.465 [b]	0.000	0.253 [c]
18y *(DTR)*	0.808 [a]	0.939 [b]	0.923 [c]	0.340 [a]	0.567 [b]	0.000	0.293 [c]
19y *(DTR)*	0.748 [a]	0.876 [b]	0.886 [c]	0.022 [a]	0.372 [b]	0.000	0.227 [c]

[a, b, c] Significant difference ($p \leq 0.05$) in the ICC between MZ and DZ twins of the same sex (or in both sexes combined)

ICC for Height (m)							
	MZ Twins			DZ Twins			
Age *(Cohorts)*	Boys	Girls	Total	Boys	Girls	Opposite-sex	Total
Birth *(All cohorts)*	0.876 [a]	0.862 [b]	0.870 [c]	0.788 [a]	0.777 [b]	0.739	0.763 [c]
5 mos *(QNTS)*	0.770	0.772	0.799 [c]	0.585	0.626	0.567	0.599 [c]
3y *(DTR)*	0.968 [a]	0.946 [b]	0.954 [c]	0.673 [a]	0.643 [b]	0.616	0.642 [c]
4y *(DTR)*	0.959 [a]	0.974 [b]	0.967 [c]	0.675 [a]	0.681 [b]	0.678	0.678 [c]
5y *(DTR & QNTS))*	0.930 [a]	0.938 [b]	0.934 [c]	0.689 [a]	0.541 [b]	0.655	0.640 [c]
6y *(DTR)*	0.970 [a]	0.977 [b]	0.974 [c]	0.638 [a]	0.591 [b]	0.604	0.614 [c]
7y *(DTR)*	0.961 [a]	0.970 [b]	0.966 [c]	0.602 [a]	0.629 [b]	0.627	0.623 [c]
8y (DTR, QNTS, & TCHAD)	0.931 [a]	0.940 [b]	0.936 [c]	0.643 [a]	0.648 [b]	0.626	0.637 [c]
9y *(CATSS & DTR)*	0.902 [a]	0.911 [b]	0.906 [c]	0.571 [a]	0.652 [b]	0.528	0.575 [c]
10y *(DTR)*	0.965 [a]	0.917 [b]	0.951 [c]	0.530 [a]	0.542 [b]	0.504	0.532 [c]
11y *(DTR)*	0.969 [a]	0.981 [b]	0.980 [c]	0.631 [a]	0.634 [b]	0.505	0.586 [c]
12y (CATSS, DTR, & BTLS)	0.890 [a]	0.917 [b]	0.905 [c]	0.493 [a]	0.588 [b]	0.497	0.523 [c]
13y *(DTR &TCHAD)*	0.933 [a]	0.925 [b]	0.932 [c]	0.549 [a]	0.565 [b]	0.422	0.504 [c]
14y *(DTR & BTLS)*	0.931 [a]	0.913 [b]	0.931 [c]	0.498 [a]	0.565 [b]	0.310	0.445 [c]
15y *(DTR)*	0.960 [a]	0.948 [b]	0.966 [c]	0.514 [a]	0.458 [b]	0.000	0.396 [c]
16y *(DTR, BTLS, & TCHAD)*	0.794 [a]	0.813 [b]	0.878 [c]	0.354 [a]	0.421 [b]	0.000	0.254 [c]
17y *(DTR)*	0.954 [a]	0.948 [b]	0.976 [c]	0.484 [a]	0.570 [b]	0.000	0.367 [c]
18y *(DTR)*	0.875 [a]	0.958 [b]	0.952 [c]	0.549 [a]	0.454 [b]	0.000	0.280 [c]
19y *(DTR)*	0.860 [a]	0.900 [b]	0.950 [c]	0.182 [a]	0.575 [b]	0.000	0.273 [c]

[a, b, c] Significant difference ($p \leq 0.05$) in the ICC between MZ and DZ twins of the same sex (or in both sexes combined)

	ICC for BMI (kg/m^2)						
	MZ Twins			DZ Twins			
Age *(Cohorts)*	Boys	Girls	Total	Boys	Girls	Opposite-sex	Total
Birth *(All cohorts)*	0.728 [a]	0.752 [b]	0.741 [c]	0.603 [a]	0.627 [b]	0.577	0.597 [c]
5 mos *(QNTS)*	0.836 [a]	0.844 [b]	0.846 [c]	0.532 [a]	0.455 [b]	0.318	0.436 [c]
3y *(DTR)*	0.883 [a]	0.906 [b]	0.897 [c]	0.584 [a]	0.639 [b]	0.225	0.403 [c]
4y *(DTR)*	0.874 [a]	0.900 [b]	0.890 [c]	0.621 [a]	0.481 [b]	0.538	0.545 [c]
5y *(DTR & QNTS))*	0.888 [a]	0.675 [b]	0.782 [c]	0.421 [a]	0.445 [b]	0.520	0.474 [c]
6y *(DTR)*	0.887 [a]	0.883 [b]	0.886 [c]	0.287 [a]	0.770 [b]	0.397	0.476 [c]
7y *(DTR)*	0.813 [a]	0.907 [b]	0.868 [c]	0.583 [a]	0.577 [b]	0.647	0.617 [c]
8y (DTR, QNTS, & TCHAD)	0.810 [a]	0.883 [b]	0.853 [c]	0.523 [a]	0.419 [b]	0.453	0.460 [c]
9y *(CATSS & DTR)*	0.884 [a]	0.848 [b]	0.870 [c]	0.493 [a]	0.467 [b]	0.406	0.444 [c]
10y *(DTR)*	0.858 [a]	0.869 [b]	0.863 [c]	0.098 [a]	0.466 [b]	0.369	0.329 [c]
11y *(DTR)*	0.899 [a]	0.913 [b]	0.910 [c]	0.631 [a]	0.571 [b]	0.344	0.502 [c]
12y (CATSS, DTR, & BTLS)	0.862 [a]	0.895 [b]	0.881 [c]	0.378 [a]	0.479 [b]	0.462	0.442 [c]
13y *(DTR &TCHAD)*	0.862 [a]	0.898 [b]	0.884 [c]	0.567 [a]	0.508 [b]	0.399	0.479 [c]
14y *(DTR & BTLS)*	0.826 [a]	0.903 [b]	0.878 [c]	0.381 [a]	0.406 [b]	0.402	0.401 [c]
15y *(DTR)*	0.857 [a]	0.921 [b]	0.904 [c]	0.513 [a]	0.654 [b]	0.276	0.487 [c]
16y *(DTR, BTLS, & TCHAD)*	0.729 [a]	0.834 [b]	0.779 [c]	0.242 [a]	0.393 [b]	0.320	0.316 [c]
17y *(DTR)*	0.873 [a]	0.937 [b]	0.915 [c]	0.057 [a]	0.477 [b]	0.000	0.157 [c]
18y *(DTR)*	0.727 [a]	0.944 [b]	0.922 [c]	0.259 [a]	0.536 [b]	0.220	0.350 [c]
19y *(DTR)*	0.769 [a]	0.793 [b]	0.789 [c]	0.067 [a]	0.355 [b]	0.276	0.294 [c]

[a, b, c] Significant difference ($p \leq 0.05$) in the ICC between MZ and DZ twins of the same sex (or in both sexes combined)
